# Factors associated with contraceptive use in Tigray, North Ethiopia

**DOI:** 10.1186/s12978-017-0281-x

**Published:** 2017-02-23

**Authors:** Araya Abrha Medhanyie, Alem Desta, Mussie Alemayehu, Tesfay Gebrehiwot, Tesfu Alemu Abraha, Atakelti Abrha, Hagos Godefay

**Affiliations:** 1 School of Public Health, Mekelle University, College of Health Sciences, Mekelle, Ethiopia; 2 UNFPA Ethiopia, Addis Ababa, Ethiopia; 3 Tigray Regional Health Bureau, Mekelle, Ethiopia

**Keywords:** Contraceptive use, Family planning, Developing countries, Ethiopia, Long acting contraceptive methods, Health extension workers

## Abstract

**Background:**

Family planning interventions are cost-effective and have several cross-cutting benefits. Despite these benefits of family planning, progress in ensuring universal access to family planning to women in developing countries has been slow. In light of this; this study investigated the prevalence and factors associated with contraceptive use in Tigray Region, Northern Ethiopia.

**Methods:**

A community-based cross-sectional study was conducted among 1966 women of reproductive age group (15–49) in 13 districts (3 urban and 10 rural) from May-June 2015. Multistage sampling technique was employed to approach the study participants. Data were analyzed using SPSS version 20. Multiple variable logistic regression analysis was used to identify the effect of independent variables on utilization of contraceptive use.

**Results:**

Out of total 1966 women, 1879 (95.6%) have ever heard about family planning. Depo-Provera (depot medroxyprogesterone acetate, or DMPA) was the most popular contractive method as mentioned by 1757 (93.5%) of the participants. The overall contraceptive prevalence rate among all women was 623 (35.6%) while the contraceptive prevalence rate among married women was 543 (41.0%). Seven-in-ten women had ever used short acting contraceptive. In fact Depo-Provera was the most common type of contraceptive used as mentioned by 402 (64.5%) of the women.

The odds of using family planning by married women living in urban areas was two times more than their counterparts (AOR = 2.0, 95% of CI: 1.33, 3.06). Similarly, the odds of using family planning among mothers with primary education was 1.3 times more as compared with no education (AOR = 1.3, 95% of CI: 1.02,1.93). However, as regards to long acting contraceptive methods, the odds of using long acting contraceptive methods use among married women in urban areas was 50% less when compared to rural married women (AOR = 0.5, 95% of CI: 0.3, 0.88).

**Conclusion:**

Contraceptive prevalence rate in Tigray region increased almost four fold in just 15 years. However, the increase is not yet enough to meet national and global targets. Further interventions are needed to narrow disparities in contraceptive use among different population groups and increase long acting contraceptive users. Moreover, improving quality of family planning in terms of the content of information provided to clients is crucial.

## Plain English Summary

Since 2003, Ethiopia has been aggressively working on improving access to basic health services including family planning to its population through a community based health program called health extension program. This community health program and other efforts by government and non-governmental organizations are believed to improve contraceptive use in the country. However, many of the studies conducted on family planning use so far employed a very small sample size and didn’t give regional representative figures. Thus, this study employed a regional representative larger sample size and assessed contraceptive use and associated factors in Tigray Region, Northern Ethiopia.

Out of total 1966 women interviewed, 1879 (95.6%) have ever heard about family planning. Injectable contraception was the most popular contraceptive method as mentioned by 1757 (93.5%) of the participants. Five hundred forty three (41.0%) of the married women participated in the study were using contraceptives during the time of interview. Seven-in-ten women had ever used contraceptives that protect pregnancy for shorter period that is three months and less. In fact injectable contraception 402 (64.5%) was the most common type of contraceptive used by the women.

Compared to figures in 2000, contraceptive use among married women in Tigray region increased almost four fold in just 15 years. However, this increase is not enough to meet national and global targets. Urban and literate women had higher chance of using contraceptive when compared to their counterparts. Further interventions are needed to narrow disparities in contraceptive use among different population groups, increase long acting contraceptive users and improve quality of family planning.

## Background

Family planning interventions are cost-effective and have several cross-cutting benefits [1]. Universal access to family planning can reduce maternal deaths by 40%, infant mortality by 10%, and childhood mortality by 21% [1–3]. However, progress in ensuring universal access to family planning to women in developing countries has been slow [3]. Globally, in 2012, about 222 million women in developing countries who desire safe, effective family planning lack access [4]. It was estimated that 54 million unintended pregnancies and 79,000 maternal deaths could have been prevented by a universal access to modern contraceptive methods [5].

Sub-Saharan African (SSA) countries have the lowest contraceptive prevalence and the highest unmet need for modern family planning, where 24% of married women have an unmet need [6–8] and only 17% of women of childbearing age use modern contraceptive methods [8]. In contrast to other regions, little or no reduction in unmet need has occurred in the past decade in Sub-Saharan Africa. Unmet need for modern methods is higher than current use (i.e., met need) in many Sub-Saharan African countries [9].

Although there has been an improvement in access to family planning services over the past two decades, contraceptive prevalence rate for Ethiopia still remains low. Contraceptive use among married women increased from 8% in 2000 to 42% in 2014 [10, 11]. However, this success is far below the national target to reach a contraceptive prevalence rate of 66% by 2015 [12]. National surveys conducted over the past 15 years showed improvement in contraceptive use in Tigray region, where this study was conducted. However, the improvement has been insufficient and remained below half of the national target [13]. Trend analysis of the Ethiopian Demographic and Health Surveys (EDHSs) data showed modern contraceptives use among married women in the region was 9.3%, 16.2%, 21.2% and 28.6% in 2000, 2005, 2011 and 2014, respectively [10, 11, 14, 15].

A study that conducted an analysis of the EDHS 2011 for geographic variation and determinants of contraceptive use showed being wealthy, more educated, being employed, higher number of living children, being in a monogamous relationship, attending community conversation, being visited by health worker at home strongly predicted use of modern contraception. While living in rural areas, older age, being in polygamous relationship, and witnessing one’s own child’s death were found to negatively influence modern contraceptive use [16]. Other studies conducted in Tigray region and other parts of Ethiopia also showed women with no history of child death, women who desire more children, women who discussed about contraceptives with their husband, women whose partners are educated, women whose partners supported the use of family planning were more likely to use contraceptives compared to their counterparts [17–19].

The finding of the mini EDHS 2014 (a CPR of 28.6%) for the Tigray region was striking and found to be lower than the CPR of other big regions of Ethiopia. Tigray region was believed to be one of the best performing regions as regards to family planning service provision however the findings of the mini EDHS was to the contrary. This lead the Tigray regional health bureau to call and initiate the present study so that to verify the findings of the mini EDHS for the region. As a result this study was conducted to determine the contraceptive prevalence rate and factors associated with it in Tigray region, north Ethiopia.

## Methods

### Study settings

A community based cross sectional study was used to assess the prevalence and factors influencing family planning use among women's reproductive age group (15–49) in Tigray region from May-June 2015. Tigray region is administratively divided into 7 zones including one especial zone, Mekelle. According to the Tigray regional health bureau report, the region has 52 districts/woredas (34 rural and 18 urban) and 792 kebeles/tabias (722 rural and 70 urban). Based on projection made from the Ethiopian census of 2007, the region had a total population of 4,806,843 of whom 2,441158 (50.8%) were female in 2015. The Region is predominantly Tegaru at 96.5% of the population and 95.6% of the population were orthodox christians. In 2014, there were 1 specialized hospital, 15 general hospitals, 7 primary hospitals, 224 health centers and 668 health posts. As regards to the number and composition of health professionals, there were 107 physicians, 3861 nurses, 778 midwives, 534 health officers and 1350 health extension workers [20].

### Sample size and sampling procedure

Using the double proportion formula for sample size determination and with the help of EPI-INFO, we calculated sample size for major variables that are assumed to affect women’s use of contraceptives. Major variables considered in the sample size calculation were residence, occupation, number of children, educational status, and desire for more children and exposure to mass media. Finally, the maximum sample size obtained was considered. During calculation, we assumed percentage of proportion of modern contraceptive acceptance by each selected major variable as taken from mini EDHS 2014 (P) - [11] -, 80% of power of study, 5% of margin of error (d), 95% confidence level, 10% non-response rate, and design effect of 2. From all alternatives, desire for more children (21.4%) and those who did not have desire (29.9%) gives the maximum sample size of 1923. The maximum sample size was taken to be the total sample size for this study. However, because of the sampling procedure used in the study, the total sample size was raised to 1966.

For the survey, 25% (13 districts/woredas) of all the districts in the Tigray region were included using systematic random sampling after making a list of the districts by alphabetical order. The list and selection of the urban and rural districts were done separately. Rural and urban districts were represented proportionally. Considering the 18:34 ratio of urban to rural districts in Tigray region and national ratio of rural population to urban population, 10 of the 13 selected districts were rural while the remaining 3 were urban districts. Accordingly *Tsegede, Medebay Zana, Adwa, Mereb Leke, Weri Leke, Gulo Mekeda, Degua Tembien, Emba Alaje, Raya Azebo* and *Kilteawelalo* districts were selected from rural districts while *Abi-Adi, Endaselalsie* and *Hawelti* districts were selected from urban districts. Similarly, using a systematic random sampling, from each selected district, 5 kebeles were selected for the study. Thus, a total of 65 kebeles were included in the study. Finally, by dividing the total sample size to the number of selected kebeles equally (1950 divided by 65), 30 households with women in reproductive age group (15–49 years) from each selected kebele were selected for interview. When two or more women of reproductive age (15–49 years) were found in a household, one was selected using the lottery method.

### Data collection procedures and data quality control

Data was collected with interview questionnaire that was adapted from EDHS questionnaire [15]. The questionnaire was pre-tested prior to the actual data collection. It contains questions regarding socio-demographic and economic characteristics, reproductive history, knowledge, attitude and practice towards utilization of family planning. It was initially prepared in English and then translated into the local language (*Tigrigna*). The interview was performed face-to-face. Eight data collectors with a health background were recruited for the data collection. To ensure data quality, training for data collectors was given. Day to day supervision of the data collection was made by the investigators of the study. A protocol that guides the design, implementation, and management of the survey was developed and given to the data collectors. Data collectors were informed and encouraged to conduct interviews in silent, comfortable places and convenient times. Besides, study participants were encouraged to give a true answer through explaining the purpose and importance of the study and assuring the confidentiality of data they are going to provide. Completed questionnaires were checked for completeness and consistency daily on the spot.

### Measurement

The study comprised and measured various variables. Variables that were operationally defined and measured were participation in incoming generating activities, presence of electronic media and being a model family or not. Participation of a woman in any income generating activity (IGA) was measured using six item questions: irrigation, cattle raring, bee keeping, poultry, garden and small scale industry. Accordingly, participation in IGA was reported and grouped into three categories: “No participation in IGA” if the woman did not participate in any income generating activity and “participation in one IGA” was considered if the woman participated in one IGA and as “participation in two or more IGAs” if a woman participated in two and more activities. The presence of electronic media was measured through the presence of one of the following electronic media: mobile, radio and television. In the Ethiopian health system, a model family is defined as a household who participate actively and implemented most of the components of the health extension program. Besides, a model family is a family which take part in different developmental activities such as agriculture, health, education and money saving activity and who show good performance in all these developmental activities. Nonetheless, in this study, we took the respondent’s word to classify whether a household is a model family or not.

### Data analysis

The collected data was entered and analyzed using SPSS Version 20. Descriptive statistics was used to summarize the data and the results were presented using frequency, tables and percentages. Binary and multiple variable logistic regression analysis were employed. Crude and adjusted odds ratios with 95% confidence intervals corresponding to the variables included in a logistic model were calculated to assess the association between explanatory variables and contraceptive use. Variables that had an association of p-vlaue less than 0.25 on binary logistic regression analysis were entered into the multiple logistic regression model analysis. In this study, a *p*-value of less than 0.05 was taken as a statistical significant value.

### Ethical issues

Ethical clearance to conduct the study was obtained from Mekelle University. Official permission was secured from Tigray regional health bureau and all district health offices included in the survey. Study participants were informed about the purpose of the study, anticipated benefits, how they are chosen to participate, data collection procedures and their full right to refuse, withdraw or completely reject part or all of the study. Participant's name was not documented or recorded. Verbal consent was obtained from each study participant.

## Result

### Socio demographic characteristics

A total of 1966 women participated in the study. The mean age of the study participants was 28.1 years  with a standard deviation (SD) of ±7.3. Of the total, 1500(76.3%) were married while the rest 197 (10%) were single, 231(11.7%) divorced and 38(1.9%) were widowed. The average number of children per woman was 3.11(SD ±1.9) and 774 (39.4%) of the respondents had 1–2 number of living children. As regards to distance from health facility, three fourth of them were found within 30 minutes of walking distance from a nearby health facility. Information regarding the educational status indicated that 849 (43.2%) of the respondents had no education. Of the total married women, 579 (38.6%) their husbands had no formal education. Seven-in-ten (69.5%) of the respondents were housewives while the remaining 600(30.5%) were either employed, merchant or student. One thousand three hundred eighty nine (70.7%) of the respondents didn't participate in any income generating activity while 269 (13.7%) women were participating in two and more income generating activities. Only 832 (42.3%) of the total respondents said that their household is a model family. Nine hundred nineteen (46.7%) respondents had radio, television or mobile phone [Table 1].

**Table 1:** Socio demographic characteristics of the study participants in Tigray region, 2015 (*N* = 1966)

Variables	N(%)
Zone	
Western	150(7.6)
North West	316(16.1)
Central	585(29.8)
Eastern	300(15.3)
South East	151(7.7)
Mekelle	150(7.6)
Southern	314(16.0)
Residence	
Rural	1516(77.1)
Urban	450(22.9)
Distance to health facility	
Up to 30 minutes	1490(75.8)
Greater than 30 minutes	476(24.2)
Age category of women	N(%)
15–19	212(10.8)
20–24	456(23.2)
25–29	487(24.8)
30–34	370(18.8)
35–39	263(13.4)
40–49	178(9.1)
Women education	
No formal education	849(43.2)
Primary education	608(30.9)
Secondary education	398(20.2)
More than secondary	111(5.6)
Husband education (*n* = 1500)	
No formal education	579(38.6)
Primary education	489(32.6)
Secondary education	274(18.3)
More than secondary	158(10.5)
Presence of electronic media (*n* = 1966)	
Presence of radio or TV or mobile phone	919(46.7)
No electronic media	1047(53.3)
Participation in IGA(*n* = 1966)	
No participation	1389(70.7)
Participation in one IGA activity	308(15.7)
Participation in 2 and more IGA activities	269(13.7)
Number of living children (*n* = 1966)	
One and less	393(23.7)
2–5	774(39.4)
3–4	496(25.2)
5 and more	378(19.2)

### Knowledge on contraceptive

Out of the total 1966 women, 1879 (95.6%) had ever heard about family planning. Of these, 1073(57.1%) heard about family planning within six months prior to the data collection. However, only 304(16.2%) of the women who had ever heard about family planning said they had heard about emergency contraceptive. Injectable contraception or *Depo-Provera* was the most popular contractive methods as mentioned by 1757 (93.5%) of the participants. As regards to source of information, health workers were the main source of information for family planning as mentioned by 702(65.4%) of the respondents. Women were asked about the benefits of FP and 1682(89.5%) of them said FP is useful for spacing births. Nine-in-ten (1676/90.2%) of the women mentioned health center, as a place for obtaining family planning [Table 2].

**Table 2:** FP methods known by women in Tigray region, 2015

Type of family planning(*n* = 1879)	Yes
	N(%)
Pill	1434(76.3)
IUCD	635(33.8)
Injection	1757(93.5)
Implanon	1292(68.8)
Jaddle	255(13.6)
Norplant	961(51.1)
Male condom	336(17.9)
Female condom	31(1.6)
Male sterilization	30(1.6)
Female sterilization	178(9.5)
Periodic abstinence	149(7.9)
Source of information about FP in the last 6 months (*n* = 1073)	
Radio	164(15.3)
Television	262(24.4)
News paper	41(3.8)
Health worker	702(65.4)
Women development army	215(20.0)
Friends	260(24.2)
HEW	590(55.0)
School	121(11.3)
Perception of women on the benefit of FP (*n* = 1879)	
To limit births	1210(64.4)
To space births	1682(89.5)
Prevent STI/HIV/AIDS	203(10.8)
As therapy	137(7.3)
To prevent unwanted pregnancy	788(41.9)
Place for FP service known by women(*n* = 1858)	
Public hospital	713(38.4)
Health center	1676(90.2)
Health post	746(40.2)
NGO health facilities	75(4.0)
Private health facilities	292(15.7)
Pharmacy	375(20.2)
Drug vendor	19(1.0)

### Contraceptive use

Of the total respondents, 132 (6.7%) women were pregnant and 1324 (67.3%) were married and non pregnant. Analysis of the findings showed 1279 (65.1%) of the total respondents had ever used a contraceptive. However, only 35 (1.8%) of the women had ever used emergency contraceptive. The overall contraceptive prevalence rate among all women was 623 (35.6%) while the contraceptive prevalence rate among married women was 543 (41.0%). Among the ever users, seven-in-ten women had ever used short acting contraceptives that is either oral contraceptive pills or Depo-Provera. In fact, Depo-Provera as mentioned by 402 (64.5%) women was the most common type of contraceptive used. Health centers were found to be the main source of contraceptives for women. Three fourth of the women (471/75.6%) received the contraceptive that they had been using during the data collection from a health center while 124 (19.9%) of the respondents said they obtained contraceptives from a health post [Table 3].

**Table 3:** Contraceptive use among women in Tigray region, 2015

Contraceptive use among all women by method (*n* = 623)	Yes
	N(%)
Contraceptive use by method among all women	
Pill	28(4.5)
IUCD	12(1.9)
Depo-Provera	402(64.5)
Implanon	158(25.4)
Jaddle	2(0.3)
Norplant	15(2.4)
Others ^a^	6(0.9)
Contraceptive use by method among married women (*n* = 540)	
Short acting	379(70.2)
Long acting	161(29.8)
Others ^b^	3(0.5)
Contraceptive use by category of method among all women	
Short acting	430(69.0)
Long acting	189(30.3)
Others ^b^	4(0.6)
Decision making on choice of methods (*n* = 623)	
Myself	558(89.6)
husband	13(2.1)
HEW	50(8.0)
Other	2(0.3)
Place where contraceptive received by all women (*n* = 623)	
Hospital	28(4.5)
Health center	471(75.6)
Health post	124(19.9)
NGO health facilities	11(1.8)
Private health facilities	4(0.6)
Pharmacy	3(0.5)
Place where contraceptive received by married women (*n* = 543)	
Hospital	23(4.2)
Health center	415(76.4)
Health post	105(19.3)
NGO health facilities	10(1.8)
Private health facilities	3(0.6)
Getting adequate information on choice of method (*n* = 623)	482(77.4)
Type of information received(*n* = 482)	
Side effect	128(26.6)
Duration of method	302(62.7)
Available method	38(7.9)
Place of service	14(2.9)

^a^Male condom, Female sterilization and Periodic abstinence

^b^Traditional and Permanent Contraceptive methods

### Contraceptive discontinuation

A total of 1279 women were asked for ever discontinuation of any type of contraceptive, out of these women, 833(65.1%) reported that they had ever discontinued contraceptive. Depo-provera or injectable contraception was the most common type of contraceptive ever discontinued by 618(74.2) women. Main reasons for discontinuation were the desire to have additional child and fear of side-effects. Five hundred ninety three (71.2%) women discontinued because they want to get pregnant and have additional child while 61(7.3%) women discontinued using a method because of fear of side-effects. Nearly two-in-ten women had ever switched from one contraceptive method to another method as a reason for discontinuation. Majority (134 /58.3%) of the women switched to Implanon [Table 4].

**Table 4:** Contraceptive discontinuation by women in Tigray region, 2015

Contraceptive discontinuation by women	Yes
	N(%)
Had you ever discontinued contraceptive use (*n* = 1279)	833(65.1)
Type of contraceptive discontinued (*n* = 833)	
Pill	83(10)
IUCD	12(1.4)
Injection	618(74.2)
Implanon	99(11.9)
Norplant	19(2.3)
Male condom	2(0.2)
Reason for discontinuation	
Want to have more children	593(71.2)
Side effect	61(7.3)
Medical problem	47(5.6)
Others^a^	10(1.3)
Partner objection	2(0.2)
Moral	1(0.1)
Fear of infertility	5(0.6)
Switch method to others	230(18.0)
Type of methods switched	
Pill	59(25.7)
IUCD	4(1.7)
Injection	134(58.3)
Implanon	26(11.3)
Norplant	7(3)
For what method have you switched	
Pill	21(9.1)
Injection	84(36.5)
Implanon	104(45.2)
Norplant	10(4.3)
Others^b^	11(4.9)
Discussion with husband about contraceptive by women within six months prior to data collection (*n* = 1422)	804(56.5)
Husband support using family planning(*n* = 1420)	1207(85)
Exclusive breastfeeding prevents pregnancy (1879)	902(48)
Up to what time exclusive breastfeeding prevents pregnancy(*n* = 902)	
Up to six months	210(22.3)
Morethan six months	553(61.3)
Don’t know	139(15.4)
Approve couple using family planning (*n* = 1679)	1548(92.2)

^a^lack of methods, partner objection, fear of infertility

^b^Female sterilization, Jaddle, IUCD, emergency contraceptive, male condom

### Attitude of women towards using contraceptives

As regards to attitude of women on using family planning, majority (nine in ten of the women) supported family planning use by couples. Out of 1422 married women, more than half (56.5%) of the women said they discussed with husband about contraceptive within six months prior to data collection. Moreover, 1207 (85%) of the women said their husband support them on using family planning [Table 4].

### Intention to use family planning methods

Out of the total, 1405 (74.8%) women had an intention to use family planning in the future. Besides, the intention to use family planning among current users was 499 (91.9%) whereas it was 531 (68%) for the non users. A significant number, 688 (49%), 1188 (84.6%) and 368 (26.2%) of the women were very confident in obtaining pill, injectable and male condom in the future, respectively. Moreover, nearly half of the respondents were very confident in obtaining Implanon whereas only 12.9% of the women were very confident in obtaining IUCD in the future. However, only 146(10%) and 23(1.6%) of the women were very confident in obtaining emergency contraceptive and female sterilization in the future, respectively.

### Contraceptive use by socio demographic characteristics

Contraceptive use had varied among the zones of the region, where the highest reported in Western zone 50(53.2%) and the least reported in South East Zone 39(33.1%). When FP utilization computed by age, younger women were more likely to use compared to older women. Thus, the highest use was reported among married women age 15–19 years (33(43.4%)) and the least 30(25%) was reported in the age group > =40 years old. It has also been found that married women with secondary education (107(47.6%)), women who had an electronic media (292(47.2%)) and women who were housewives in occupation (422(40.8%)) were the most users of family planning methods. Besides married women whose husband education is secondary (112(47.3%)), women who were participating in one income generating activities (92(42.6%)) and women who had one or no live children were reported as more current users of family planning. Of those FP users among married women, (237(39.9%)) were members of model family [Table 5].

**Table 5:** Current contraceptive prevalence rate among married and non pregnant women by socio demographic variables (*N* = 1324)

Variables	Noncurrent user N (%)	Current users N (%)	Total N (%)
Occupation status of women)			
Housewife	632(60.0)	422(40.0)	1054(100)
Others^a^	149(55.2)	121(44.8)	270(100)
Women education			
No formal education	388(65.9)	201(34.1)	589(100)
Primary education	241(55)	197(45)	438(100)
Secondary education	118(52.4)	107(47.6)	225(100)
More than secondary	34(47.2)	38(52.8)	72(100)
Husband education			
No formal education	316(62.7)	188(37.3)	504(100)
Primary education	266(61)	170(39)	436(100)
Secondary education	125(52.7)	112(47.3)	237(100)
More than secondary	74(50.3)	73(49.7)	147(100)
Presence of electronic media			
Presence of radio or TV or mobile phone	327(52.8)	292(47.2)	619(100)
No electronic media	454(64.4)	251(35.6)	705(100)
Participation in IGA			
No participation	515(57)	388(43)	903(100)
Participation in one IGA activity	124(57.4)	92(42.6)	216(100)
Participation in 2 and more IGA activities	142(69.3)	63(30.7)	205(100)
Model family			
Yes	357(60.1)	237(39.9)	594(100)
No	424(58.1)	306(41.9)	730(100)
Number of living children			
One and less	150(52.8)	134(47.2)	284(100)
2–5	453(58.9)	316(41.1)	769(100)
Six and more	143(69.1)	64(30.9)	1260(100)

^a^(farmer, employee, merchant and student)

### Factors affecting contraceptive use

Adjusting for other variables; difference in contraceptive use by the zone in which a woman lives became significant. Women who live in North West zone were 60% less likely (AOR = 0.4, 95% of CI 0.27, 0.81), central zone 50% less likely (AOR = 0.5, 95% of CI 0.29, 0.97) and Eastern zone was 50% less likely (AOR = 0.5, 95% of CI 0.27, 0.99) as compared with women who live in Western zone. Moreover, the odds of using family planning was 61% less in South East zone (AOR = 0.39, 95% CI 0.18, 0.83) and 60% less in Southern zone (AOR = 0.4 95% of CI 0.26, 0.86) as compared with Western zone.

Place of residence (urban versus rural) was found to be a significant predictor of contraceptive use. The odds of using contraceptive in the urban area was two times more in married women than their counterparts (AOR = 2.0, 95% of CI: 1.33, 3.06). Women education also contributes to enhancing the utilization of contraceptives. As such, the odds of using a family planning among mothers with primary education was 1.3 times more as compared with no education (AOR = 1.3, 95% of CI: 1.02,1.93)

The odds of using contraceptive among women who had discussion with their husband about family planning was three times more as compared with their counterparts (AOR = 2.9, 95% CI: 2.2,3.85). Women who knew three and more contraceptive methods had increased chance of using family planning (AOR = 1.6, 95% CI = 1.21, 2.27) as compared with women who knew two and less contraceptive [Table 6].

**Table 6:** Predictors of Family planning use by women in Tigray region, 2015 (*N* = 1324)

Variables	Family planning use		COR[95%]	AOR[95%]
	Yes	No		
	N (%)	N (%)		
Zone				
Western	50(53.2)	44(46.8)	1	1
North West	106(46.1)	124(53.9)	0.7(0.46,1.21)	0.4(0.27,0.81)*
Central	149(37.8)	245(62.2)	0.5(0.34,0.84)*	0.5(0.29,0.97)*
Eastern	67(36.4)	117(63.6)	0.5(0.3,0.83)*	0.5(0.27,0.99)*
South East	39(33.1)	79(66.9)	0.4(0.24,0.75)*	0.39(0.18,0.83)*
Mekelle	53(51.5)	50(48.5)	0.9(0.53,1.62)	0.8(0.49.1.57)
Southern	79(39.3)	122(60.7)	0.5(0.34,0.93)*	0.4(0.26,0.86)*
Residence				
Rural	377(37.3)	634(62.7)	1	1
Urban	166(53.0)	147(47.0)	1.8(1.47,2.45)	2.0(1.33,3.06)*
Women education				
No formal education	201(34.1)	388(65.9)	1	1
Primary education	197(45)	241(55)	1.5(1.22,2.03)	1.3(1.02,1.93)*
Secondary education	107(47.6)	118(52.4)	1.7(1.28,2.39)	1.0(0.70,1.64)
More than secondary	38(52.8)	34(47.2)	2.1(1.31,3.53)	1.0(0.54,1.97)
Number of living children				
One and less	134(47.2)	150(52.8)	1	1
2–5	316(41.1)	143(69.1)	0.7(0.59,1.02)	0.8(0.54,1.19)
Six and more	64(30.9)	453(58.9)	0.5(0.34,072)	0.8(0.45,1.56)
Number of family planning methods known by women				
Up to two	106(29.9)	248(70.1)	1	1
Three and above	437(45.3)	528(54.7)	1.9(1.49,2.51)*	1.6(1.21,2.27)*
Distance to health facility				
Up to 30 minutes	433(42.3)	591(57.7)	1.2(0.97,1.65)	2.6(0.21,2.37)
Greater than 30 minutes	110(36.7)	190(62.3)	1	1
Discuss with husband about family planning use				
No	143(25.2)	424(74.8)	1	1
Yes	398(54.4)	333(45.6)	3.5(2.79,4.5)*	2.9(2.20,3.85)*
Approve couples using family planning				
No	20(27.4)	53(72.6)	1	1
Yes	475(44.3)	597(55.7)	2.1(1.24,3.57)	1.4(0.78,2.7)

*P <0.05 = Significant

This study also analyzed the predictors of utilization of long acting contraceptive methods. The findings of the analysis showed place of residence (urban versus rural) of married women was found to be a significant predictor of long acting family planning use. The odds of using long acting family planning methods among married women in the urban area was 50% less when compared to rural married women (AOR = 0.5, 95% of CI: 0.3, 0.88) [Table 7].

**Table 7:** Predictors of long acting family planning use in Tigray region, 2015 (*N* = 540)

Variables	Family planning use		COR[95%]	AOR[95%]
	Yes	No		
	N (%)	N (%)		
Residence			1	1
Rural	249(66.2)	127(33.8)	1	0 .5(0.30,0.88)
Urban	130(79.3)	34(20.7)	0.5(0.33,0.79)*	
Women education				
No formal education	144(71.6)	57(28.4)	1	1
Primary education	129(65.8)	67(34.2)	1.3(0.85,2.0)	1.4(0.88,2.40)
Secondary education	73(69.5)	32(30.5)	1.1(0.66,1.85)	1.6(0.85,3.22)
More than secondary	33(86.8)	5(13.2)	0.3(0.14,1.02)	0.4(0.1,1.64)
Number of family planning methods known by women				
Up to 2	81(76.4)	25(23.6)	1	1
Three and more	298(68.7)	136(31.3)	1.4(0.9,2.4)	1.3(0.82,2.34)
Approve couples using family planning				
No	10(50)	10(50)	1	1
Yes	338(71.5)	135(28.5)	0.3(0.16,0.98)	0.4(0.17,1.10
Presence of electronic media (*n* = 540)				
No electronic media	166(66.1)	85(33.9)	1	1
Presence of radio or TV or mobile phone	213(66.1)	85(33.9)	0.6(0.48,1.0)	0.8(0.52,1.33)
Husband education				
No formal education	132(70.2)	56(29.8)	1	
Primary education	107(63.3)	62(36.7)	1.3(0.87,2.12)	1.3(0.8,20)
Secondary education	84(75.7)	27(24.3)	0.7(0.44,1.29)	0.9(0.48,1.72)
More than secondary	56(77.8)	16(22.2)	0.6(0.35,1.27)	0.9(0.40,2.20)

*P <0.05 = Significant

## Discussion

The overall contraceptive prevalence rate among all women was 35.6% while it was 41.0% among married women. Zonal distribution, residence, educational status of women, having discussion with husband and number of family planning methods known by women were significant predictors of modern contraceptive use among married women while residence was the potent predictor of long acting contraceptives use. This study showed that the overall contraceptive prevalence rate for Tigray region was 41.0% among married women. Comparison of this finding with findings of previous studies shows family planning use in Tigray region is steadily increasing. In just fifteen years, contraceptive prevalence rate in the region increased from 9.3% in 2000 to 41% in 2015. Trend analysis of the Ethiopian Demographic and Health Surveys data showed modern contraceptives use among married women in the region was 9.3%, 16.2%, 21.2% and 28.6% in 2000, 2005, 2011 and 2014, respectively [10, 11, 14, 15]. This might be attributed to the coordinated efforts made by the Ethiopian governments and its partners in particular through the health extension program. Since 2003, a total of about 38,000 health extension workers (HEWs) had been trained and deployed throughout the country. HEWs are trained for one year. They educate and provide basic health services include family planning. The number of health centers throughout the country increased by 519% between 2004 and 2010. This has resulted in remarkable increase in health service coverage [12, 21]. Besides, the universal increase in knowledge of family planning among women as identified in this study and in a recent surveys may also contribute to this fourfold increase. According to the present study, knowledge measured by hearing at least one method of contraceptive is universal (95.6%). This is consistent with the findings of the EDHS which showed a national coverage of women’s knowledge about family planning methods (96.7%) [11]. Nevertheless, a 41% of CPR is still lower than the national CPR target set for reaching a CPR of 66% by 2015 [12]. Hence, innovative and rigorous interventions are still needed to meet the desired goals.

The findings of this study showed that the composition of short acting versus long acting contraceptive use in the region was 70% to 30%. This might imply most of the women still preferred short acting contraceptives and the government should strengthen the method mix of the family planning and continuous awareness creation on the importance of long acting family planning, since they are cheap, highly effective and need less visits to health institution. Besides, the low uptake of long acting contraceptive methods could be attributed to lack of trained health professionals, inadequate counseling on the methods and lack of method mix. Thus, the Ethiopian health system should address these possible gaps.

Although the use of long acting contraceptives is still low, paradoxically the proportion of women using long acting contraceptives in rural areas is higher than the urban areas [22]. This might be due to the task shifting intervention made by the Ethiopia Federal Ministry of Health. With this task shifting approach Implanon insertion which has been previously given only by health workers at health center or higher level of health facility is also currently given by trained health extension workers at health posts [23]. This approach allows rural women to have access to Implanon insertion service from health extension workers at health posts.

Despite the increase in the overall CPR, this study showed there were disparities in the proportion of contraceptive use among different population groups by location (Zone), residence (Urban Vs Rural). For instance, in the study, the odds of using any modern contraceptives among married women in urban areas was two times more in married than rural women. However, it was the opposite when it comes to using long acting contraceptives. The proportion of long acting contraceptive users was significantly higher in rural areas as compared with urban areas. This finding is consistent with findings of a similar study conducted in the Tigray region [22]. This might indicate difference in the performance as well as the approach to address the issue of family planning from place to place. Thus, the health system should try to capture the best practice in well performing areas so as to share these best experiences to less performing areas. Besides, further study is necessary to investigate the actual reasons for these observed disparities.

This study revealed that the odds of using family planning among women who had discussions with their husband about family planning were higher as compared with their counterparts. Similarly, a study done in Debremarkos, Ethiopia revealed that mothers who had discussions with their husband were more likely to use family planning than their counterparts [24]. This indicates that male involvement in family planning could be a factor in increasing the number of family planning users. This implies family planning programs should consider involving men as part of their intervention to enhance the proportion of family planning users.

Having information on the discontinuation and switching of family planning is essential for policy maker, program manager and implementer. Nearly two third women ever discontinued any type of contraceptive and injection was the most common type of method ever discontinued. Besides, out of the total study participants, nearly sixty percent women switched to implanon. This implies a lot of women got counseling on the importance of implanon which is currently provided at the grassroots level by the HEWs [23].

Informed choice is an important principle in the delivery of family planning services. As an aspect of informed choice, it is required that all family planning providers inform users about potential side effects of the method and what they should do if they encounter such side effects. This information assists the user in coping with side effects and thus decreases discontinuation of temporary methods. Contraceptive users should also be informed of the other methods available to them [15]. Even though eight-in-ten of the women who participated in this study got adequate information on the choice of method, only one fourth of the study participants got information about side effect. This calls for health providers to provide adequate and comprehensive information to clients during FP counseling sessions.

Compared to previous studies on Family planning in the region, this study takes larger sample size which is more representative [22, 25, 26]. Thirteen (25%) of the districts in the region are included in the survey and both urban and rural areas are represented. However, the findings of this study might be limited by the fact that women living in very remote areas with no access to transportation or far from an hour walking distance from the center of the selected villages (kebeles) were not reached. Besides, in this study, we did not analyze the women’s income along with other independent variables and unmet need for family planning. The data gathered on these variables had problems and thus excluded from the analysis during the data cleaning process.

## Conclusion

Contraceptive prevalence rate in Tigray region increased almost four fold in just 15 years. This might be attributed to the strong political commitment and increased community mobilization and service provision through the health extension program. However, much effort is still needed to meet national target which was set to reach a contraceptive prevalence rate of 66% by 2015.

Despite the increase in overall CPR, this study revealed disparities in contraceptive use among different population groups and poor quality of family planning services in terms of content of information provided to clients. Hence, the federal ministry of health and its partners should devise a mechanism to narrow the observed disparities in contraceptive use and improve quality of FP services.

## References

[1] Family Care International: Save motherhood review. The Safe Motherhood Initiative 1987–2005. New York: Family Care International, Inc.; 2007.

[2] Smith R, Lori A, Jay G, Donna C. Family planning saves lives. 4th ed. Washington: Population Reference Bureau; 2009. http://www.prb.org/Publications/Reports/2009/fpsl.aspx. Accessed 6 Feb 2017.

[3] USAID (2009). Update on family planning in Sub-Saharan Africa. Repositioning family planning: guidelines for advocacy action.

[4] Singh S, Darroch JE (2012). Adding It Up: Costs and Benefits of Contraceptive Services—Estimates for 2012.

[5] Darroch JE, Sedgh G, Ball H (2011). Contraceptive technologies: responding to women's needs.

[6] United Nations Department of Economic and Social Affairs. What would it take to accelerate fertility decline in the least developed countries? UN Population Division Policy Brief No. 2009/1, Mar 2009

[7] United Nations. World population monitoring, focusing on the contribution of the program of action of the international conference on population and development of the internationally agreed development goals, including the millennium development goals. Report of the Secretary-General (E/CN.9/2009/3). 2009

[8] WHO, UNICEF, UNFPA, World Bank. Maternal mortality estimates in 2005. WHO: Geneva, Switzerland.2007, ISBN 978 92 4 159621 3.>

[9] Jacob Stein R, Bakamjian L, Pile JM (2008). Threatened and still greatly needed Family planning programs in Sub-Saharan Africa.

[10] Central Statistical Agency (Ethiopia) and ORC Macro (2000). Ethiopia Demographic and Health Survey 2000.

[11] Central Statistical Agency [Ethiopia] and ICF International (2014). Ethiopia Demographic and Health Survey 2014.

[12] Federal Ministry of Health of Ethiopia (2010). Health Sector Development Program IV (2010/11-2014/15).

[13] Medhanyie A, Spigt M, Kifle Y, Schaay N, Sanders D, Blanco R, GeertJan D, Berhane Y (2012). The role of health extension workers in improving utilization of maternal health services in rural areas in Ethiopia: a cross sectional study. BMC Health Serv Res..

[14] Central Statistical Agency (Ethiopia) and ORC Macro (2006). Ethiopia Demographic and Health Survey 2005.

[15] Central Statistical Agency [Ethiopia] and ORC Macro (2011). Ethiopia Demographic and Health Survey.

[16] Lakew Y, Reda A, Tamene H, Benedict S, Deribe K (2013). Geographical Variation and Factors Influencing Modern Contraceptive Use among Married Women in EthiopiaEvidence from a National Population Based Survey. Reprod Health.

[17] Mekonnen W, Worku A (2011). Determinants of Low Family Planning Use and High Unmet Need in Butajira District. South Central Ethiopia. Reprod Health.

[18] Mohammed A, Woldeyohannes W, Feleke A, Megabiaw B (2014). Determinants of Modern Contraceptive Utilization among Married Women ofReproductive Age Group in North Shoa Zone, Amhara Region, Ethiopia. Reprod Health.

[19] Gebremariam A, Addissie A (2014). Intention to use long acting and permanent contraceptive methods and factors affectingit among married women in Adigrat town, Tigray, Northern Ethiopia. Reprod Health.

[20] Tigray Regional Health Bureau (2014). Annual Profile 2014.

[21] Federal Ministry of Health of Ethiopia (2010). Health Sector Development Programme III : Annual performance report.

[22] Alemayehu M, Aster K, Alem D, Hailay G, Tesfalem H, Henock Y (2015). Rural women are more likely to use long acting contraceptive in Tigray region. Northern Ethiopia: a comparative community-based cross sectional study. BMC Women's Health..

[23] Federal Ministry of Health of Ethiopia: National Guideline for Family Planning Services in Ethiopia. Addis Ababa: Ministry of Health; 2011.

[24] Gizachew AB, Tatek AZ, Teresa KB (2014). Demand for long acting and permanent contraceptive methods and associated factors among married women of reproductive age group in Debre Markos Town. North West Ethiopia. BMC Women’s health..

[25] Alemayehu M, Belachew T, Tilahune T (2012). Factors affecting utilization of long acting and permanent contraceptive among married women of reproductive age group in Mekelle Town. BMC Pregnancy Child Birth..

[26] Alemayehu M, Etana B, Fisseha G, Haileslassie K, Berhe Y, Henock Y (2015). Integration of long acting and permanent contraceptive methods with an ART program was poor in Tigray Region, Ethiopia. Global J Med Res E Gynecol Obstet.

